# Salience of spatiochromatic patterns

**DOI:** 10.1167/jov.21.4.7

**Published:** 2021-04-16

**Authors:** Amanda C. Hardman, Jasna Martinovic

**Affiliations:** 1 School of Psychology, University of Aberdeen, Aberdeen, UK; 2 Department of Psychology, School of Philosophy, Psychology and Language Sciences, University of Edinburgh, Edinburgh, UK

**Keywords:** color, luminance, salience, perceived contrast, spatiochromatic processing, single-opponent neurons, double-opponent neurons

## Abstract

We investigated the dependence of perceived contrast on cone-opponent stimulus content and its spatial distribution. Participants matched a comparison patch to a light gray standard of fixed contrast. The first experiment determined the point of iso-salience for gratings, Gabors and Gaussians along cardinal directions in cone-opponent color space for two-alternative forced choice (2AFC) and adjustment tasks. No difference was found between adjustment and 2AFC tasks, meaning that adjustment tasks provide a quick and robust way to measure perceived contrast, at least for relatively large suprathreshold stimuli. In line with the differences in contrast energy between Gaussians, Gabors, and gratings, Gaussians required less contrast to achieve equal perceived salience with a standard irrespective of color. More surprisingly, bluish Gaussians were found to have higher salience than yellowish Gaussians at equal levels of contrast. Although perceived contrast of grating and Gabor patterns likely depends on spatial frequency channels that at 1 cycle-per-degree are not too dissimilarly tuned for color and luminance, for Gaussians the contribution of single-opponent neurons would be greater for color than for luminance. In a follow-up experiment, we found that the bluish/yellowish asymmetry decreased as we reduced the proportion of the lowpass non-flat contrast distribution in the stimulus, with minimal asymmetry for the stimulus with a flat contrast distribution (i.e., uniform patch). Combined, this means that differential engagement of spatial frequency channels, single-opponent and double-opponent neurons impacts on perceived contrast of chromatic suprathreshold stimuli. Perceived contrast thus provides a window into neural computations enacted by low-level cone-opponent mechanisms.

## Introduction

Controlling for the various physical features of one's stimuli is a major issue in vision research. This is particularly true of research interested in the processing of salience. Salience, as reflected in perceived contrast, has been shown to vary depending on the physical features of a stimulus. Spatial frequency, for example, affects perceived contrast when physical contrasts approach participants’ detection thresholds (i.e., below ∼20% Michelson contrast; Georgeson and Sullivan, 1975). In fact, for such relatively low-contrast stimuli, perceived contrast (i.e., perceived salience) depends on detection thresholds (Kulikowski, 1976; Georgeson, 1991).

Researchers often equate stimulus salience between color and luminance by using the same multiple of their detection threshold (for a discussion, see Shevell & Kingdom, 2008). However, there has been some evidence to suggest that using multiples of detection thresholds to equate perceived contrast of stimuli is not ideal (Sally & Gurnsey, 2004; Gurnsey, Sally, & Ball, 2002). Perceived contrast is usually equated by subtracting the detection threshold from physical contrast. This can be done either linearly (Kulikowski, 1976; McIlhagga, 2004) or on a log scale (Peli, Young, Goldstein, & Reeves, 1991). The log scale method seems to be more effective at low luminance levels (below 8 cd/m^2^). As described by McIlhagga (2004), the equation for perceived contrast (P) would be P = C + *e*/g – *f*/*g*, where C is the physical contrast, *g* is the gain or attenuation of the contrast signal, *e* is the noise in the system, and *f* is the threshold value for generating a response within the neuron. In the case of small levels of noise threshold T = *f*/*g* and *e*/*g* approaches 0, meaning P = C – T. This justifies Kulikowski's approach of calculating perceived contrast as a linear subtraction of the detection threshold from the physical contrast of a stimulus. In fact, McIlhagga's (2004) formula corresponds to the earlier proposal by Barlow that detection thresholds represent a good estimate of neuronal noise (Barlow, Kaushal, Hawken, & Parker, 1987).

According to our knowledge, Switkes and Crognale (1999) were the first to compare perceived contrast between chromatic and achromatic stimuli. They found that participants matched perceived contrast between them with reasonable intraobserver and interobserver consistency. Moreover, matches of color and luminance stimuli were found to be transitive. Subsequently, Switkes (2008) reported certain asymmetries in perceived salience for both L − M and S− (L + M) cone-opponent mechanisms. They used a two-alternative forced-choice (2AFC) task to determine which of two one-cycle-per-degree (cpd) unipolar horizontal gratings was perceived as more salient by participants. Varying the contrast of gratings between complementary chromaticities (i.e., one grating was reddish, the other greenish) enabled comparisons of the perceived contrast of opposing color polarities. Through this method, it was found that participants required more contrast for greenish compared to reddish and for bluish compared to yellowish to achieve equal perceived salience between colors. In other words, for participants to perceive bluish and yellowish gratings as being of equal contrast, the physical contrast of the bluish gratings had to be higher than the physical contrast of the yellowish gratings.

A possible reason for the bluish/yellowish asymmetry in perceived contrast found by Switkes is the difference in neuronal behavior between S+ (“blue-on”) and S− (“blue-off”) cells, which suggests different pathways for S increments and decrements (see de Monasterio, 1979 and Tailby, Solomon, & Lennie, 2008). Although many studies have found an asymmetry between S+ and S− responses, either through neuronal responses or detection or discrimination thresholds, opinions are divided on the direction of this asymmetry. Tailby, Solomon, and Lennie (2008) and Tailby, Szmajda, Buzás, Lee, and Martin (2008), for example, found differences in response saturations, contrast gains (i.e., increases in firing rate per unit of contrast) and spatial frequency sensitivities between LGN S+ and S− neurons. LGN S+ neurons were more sensitive to S cone modulation at mid-levels of contrast, less sensitive to luminance modulation, had response saturations at higher contrasts and had higher contrast gains (i.e. increases in firing rate per unit of contrast) compared to S- neurons. However, the S+/S− asymmetry in firing rates varied depending on the spatial frequency used: S+ neurons had higher firing rates for 0.3 cycles per degree stimuli compared to lower spatial frequencies. In comparison, S− neurons were more sensitive (i.e., had higher firing rates) to uniform fields compared to the 0.3 cycles per degree stimuli optimal for S+ neurons. This difference in spatial frequency sensitivity resulted in S+ and S− neurons having approximately equal firing rates when presented with a large (10°) S− cone-selective grating. Vingrys and Mahon's (1998) discrimination results are in line with Switkes’ (2008) findings that bluish required more contrast than yellowish to achieve equal salience between the two. Their detection threshold results, on the other hand, disagree with Sakurai and Mullen's (2006) findings that bluish and yellowish have equal detection thresholds. Perhaps the differences in results for these studies can be explained by the different stimuli used, as well as the level of contrast used in each experiment: Sakurai and Mullen (2006) used small elongated Gaussian blobs at the level of detection thresholds, Switkes (2008) used suprathreshold gratings, Tailby, Solomon and Lennie (2008) and Tailby, Szmajda, et al. (2008) used suprathreshold full field stimuli and gratings, Vingrys and Mahon (1998) used small spots (uniform 1° circular patches) at detection threshold and suprathreshold levels of contrast. Because perceived salience has been shown to differ not only between different spatial patterns, but also between different colors, care must be taken when generalizing the findings on perceived salience of color obtained from vastly different stimulus types.

Georgeson (1991) proposes that contrast encoding depends largely on the response amplitude of the most active spatial filter. Switkes (2008) compared chromatic gratings in the 0.25 to 2 cpd range. Depending on the spatial frequency content of the presented stimulus, distinct neuronal populations would be activated. Single-opponent neurons are preferentially activated by equiluminant uniform or low spatial frequency patterns whereas double-opponent neurons are activated by higher spatial frequencies and the presence of edges (Nunez, Shapley, & Gordon, 2018; Thorell, de Valois, & Albrecht, 1984). These two neuronal populations not only vary in their activity across different spatial frequencies but also differ in their responses to stimuli defined using the two cone-opponent (L−M [reddish/greenish] and S−(L+M) [bluish/yellowish]) color mechanisms (Solomon, Lee, White, Rüttiger, & Martin, 2005; Thorell, de Valois, & Albrecht, 1984). Thus directly comparing the results of studies that use different stimuli is problematic, because the stimuli may activate separate neuronal populations.

To fill these gaps in knowledge, this study aimed to determine whether changing the spatial pattern of a stimulus affected the asymmetries in perceived contrast between poles of cone-opponent mechanisms on a much larger sample of participants and with two different tasks (adjustment and 2AFC). Although it is obvious that the difference in the amount and spatial distribution of contrast energy between, for example, gratings and Gaussians will inevitably lead to differences in perceived contrast between these patterns, the key question is whether differences will emerge for any of these pattern types between the two poles of cone-opponent mechanisms. In particular, we aimed to establish whether bluish would still require more physical contrast to achieve equal perceived contrast with yellowish for patterns other than gratings (as in Switkes, 2008). This curiosity was at least in part driven by our observation of the opposite result (bluish more salient than yellowish) in a study in which we used salience matching to equate the appearance of small bluish or yellowish Gaussian patches to a light gray standard (Hardman, Töllner, & Martinovic, 2020).

## General methods

### Participants

All participants had normal or corrected-to-normal vision. The Cambridge Color Test was used to check for any color vision deficiencies (Regan, Reffin, & Mollon, 1994). Participants gave their written informed consent and were reimbursed for their time and effort. The experiment was approved by the University of Aberdeen's Psychology Ethics Committee and was in accordance with the Declaration of Helsinki.

### Color space and experimental colors

Because these experiments were interested in comparing the perceived contrasts of cone-opponent and cone-additive colors, all stimuli were defined using the DKL color space (Derrington, Krauskopf, & Lennie, 1984). The cardinal axes of DKL color space isolate the responses of the L − M, S − (L + M) and L + M color mechanisms. The six cardinal DKL axis colors are increments and decrements along these axes. The increments and decrements that isolate the L − M and S − (L + M) cone-opponent mechanisms and the increments and decrements that isolate the L + M cone-additive mechanism will be referred to as reddish/greenish, bluish/yellowish, and light gray/dark gray, respectively. Because our stimuli involve unidirectional modulations of contrast, we calculate contrast of the maximal value within the stimulus (e.g., peak of the Gaussian) relative to the background. We first calculate Weber cone contrast for ΔL, ΔM, and ΔS, using the method outlined in Golz and MacLeod (2003). We use these values to calculate contrasts within each mechanism.

### Stimuli

**Figure 1.**
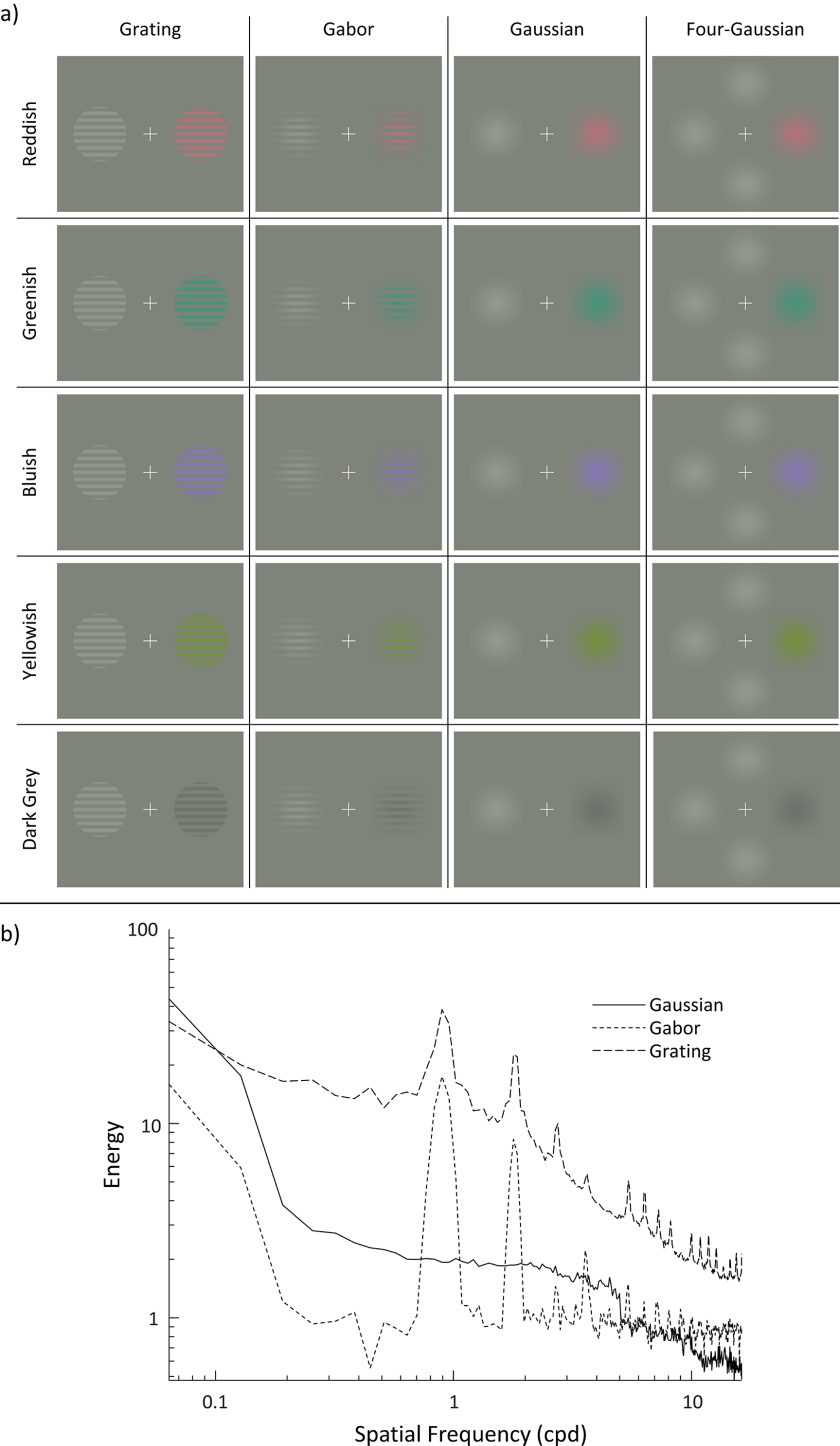
The spatial patterns and five experimental colors used in Experiment 1 are shown in panel (a). The columns show the four spatial patterns (grating, Gabor, Gaussian, and four-Gaussian); the rows show the five experimental colors (reddish, greenish, bluish, yellowish, and dark gray) with their fixed light gray standard. Although the experimental color could be displayed either left or right of the fixation cross during the experiment, only those conditions with the experimental color on the right are shown in this example. Panel (b) shows the contrast magnitudes of the grating, Gabor, and Gaussian stimuli plotted as a function of their spatial frequencies. Note the graph is plotted on a log-log scale.

All stimulus patches used in this experiment had radii of 5° of visual angle, and their center was located 6.5° away from a centralized fixation cross (Figure 1).

### Apparatus

Both experiments were run on a DELL PC equipped with a dedicated visual stimulus generator (ViSaGe; Cambridge Research Systems, Ltd., Kent, UK) and presented on an Ilyama Vision Master Pro 450 CRT monitor. Stimulus presentation was controlled using Matlab (MathWorks, Natick, MA, USA). All staircase procedures used in the experiments were implemented using the Palamedes Toolbox for Matlab (Kingdom & Prins 2010). The chromatic and luminance output of the monitor were calibrated using the CRS calibration system (ColorCAL; Cambridge Research Systems, Ltd.); the accuracy of the calibration was verified with a spectroradiometer (SpectroCAL; Cambridge Research Systems, Ltd.). The monitor had been switched on for at least 30 minutes before any experiment. Participants’ viewing distance was 70 cm. A Cedrus R530 (Cedrus Corporation; San Pedro, CA, USA) button box was used to collect participant responses. All stimuli were presented on a neutral background corresponding to the DKL white point. The brightness of the screen varied slightly between Experiments 1 and 2: the white point was set to CIE 1931 0.2998, 0.3107, 46.64 cd/m^2^ in Experiment 1 and CIE 1931 0.3006, 0.3125, 46.71 cd/m^2^ in Experiment 2.

### Luminance adjustment: heterochromatic flicker photometry

Because of individual differences in isoluminance points of opposing colors (e.g., 0˚ and 180˚ in DKL color space), luminance confounds may be present in supposedly isoluminant color stimuli. To remove these luminance confounds from color-isolating stimuli, opposing colors were adjusted to isoluminance for each participant using heterochromatic flicker photometry (HCFP; Walsh, 1958). The stimulus display (which matched the experimental stimuli described in individual experiments) flickered between opposing colors at a rate of 20 Hz.

Participants were instructed to adjust the luminance of the stimuli (using the left and right buttons on the button box) until a minimum amount of flicker was perceived. At this point, the two colors were isoluminant.

Eight trials were recorded per condition. The trials with the highest and lowest values were then discarded, and the averages from the remaining six trials used to adjust experimental stimuli to isoluminance. Separate HCFP was performed for each spatial pattern used.

### Contrast adjustment task

For this task, participants altered the contrast of an adjustable “target” patch (one of a set of experimental colors) until the perceived contrast of this “target” patch matched the perceived contrast of a fixed-contrast light gray “standard.” Contrast adjustments to the “target” stimulus were made using the left (increase contrast) and right (decrease contrast) buttons of the button box.

Twenty trials were performed per condition. All trials for all conditions for a certain spatial pattern were randomized and run together. Participants were given a break halfway through. Thus the length and number of the adjustment sets performed varied between experiments (see relevant individual methods sections).

## Experiment 1—perceived contrast and spatial stimulus properties

### Materials and methods

#### Participants

Nineteen participants (12 female; 18 right-handed; age range 19-58, *M* = 25, *SD* = 10, one outlier at 58 years old) performed the contrast adjustment task. Fifteen of these participants also took part in additional two-alternative forced-choice and detection threshold tasks (see Experiment 1: Tasks).

#### Stimuli

To determine whether perceived contrast varied between opposing color polarities in DKL space, the experiment used the six cardinal colors (reddish, greenish, bluish, yellowish, light gray, and dark gray). Switkes (2008) previously demonstrated that pairwise contrast comparisons were transitive. That is, the results of a pairwise contrast match could be predicted using the results of two pairwise contrast matches between the two colors of interest and a common standard. For example, a pairwise match between reddish and greenish could be predicted using contrast matches of reddish to light gray and greenish to light gray. It was therefore concluded that (1) perceived contrast of experimental colors could be indirectly compared to each other by performing contrast matches against a standard and (2) the results of the contrast matches would not be affected by the designated color of the standard. All contrast comparisons were therefore performed against a light gray standard. The other five colors (reddish, greenish, bluish, yellowish, and dark gray) were the experimental colors, matched by participants in terms of perceived contrast with the standard. The light gray standard had a Weber contrast of 0.17 at the point of its maximal contrast. When recalculated as the root of the sum of the squared cone contrasts (rms contrast), our light gray standard had a contrast of 0.36—this is more than twice that of Switkes’ study.

To compare different spatial patterns, gratings, Gabors and Gaussians were generated for each of the six colors. Visual displays consisted of two patches (varying only in color) left and right of a centered fixation cross. One of the two patches was the light gray standard. The other was one of the five experimental colors. Gratings and Gabors had spatial frequencies of one cycle per degree. Gabors and Gaussians had Gaussian deviations of 22.4% of patch size. All patches had radii of 5° visual angle.

The effect of stimulus configuration was also tested by generating a four-patch condition using the same display as the Gaussian condition, with the addition of two Gaussians (of the same size and deviation) 6.5° above and below the centered fixation cross. In this case, three of the patches were the light gray standard and one was one of the five experimental colors. This condition will be referred to as “four-Gaussian.”

Examples of the four spatial patterns (for each of the five experimental colors) used in this experiment are shown in Figure 1a. To enable comparison of the contrast energy and spatial frequency content of the three main spatial patterns (gratings, Gabors and Gaussians), contrast magnitude is plotted as a function of spatial frequency in Figure 1b.

#### Luminance adjustment: heterochromatic flicker photometry

As reddish, greenish, bluish and yellowish were used in this experiment, the HCFP was performed for both reddish/greenish and bluish/yellowish conditions. Participants completed all trials for the bluish/yellowish condition, followed by all trials for the reddish/greenish condition. The step sizes were ±0.008 Weber luminance contrast for the bluish/yellowish condition and ±0.014 Weber luminance contrast for the reddish/greenish condition. The degree of elevation displayed at the beginning of each trial for each condition was randomized (trial-by-trial) within the range of 0 ±0.041 for the bluish/yellowish condition and ±0.070 for the reddish/greenish condition. The level of individual variation is much higher for the L-M mechanism, thus the higher range. Because there were four spatial patterns (grating, Gabor, Gaussian, and four-Gaussian), the HFCP procedure was performed for each spatial pattern, as well as each pair of colors.

#### Tasks

The effect of different tasks was determined by measuring the point of equal perceived contrast using both a contrast adjustment task and a standard two-alternative forced-choice task (2AFC). Order of the tasks was randomized across participants.

##### Contrast adjustment

Participants performed adjustment sets using the five experimental colors (reddish, greenish, bluish, yellowish, dark gray) and four spatial patterns. Each color had equal initial contrasts and step sizes for the four spatial patterns. Reddish grating, Gabor, Gaussian and four-Gaussian conditions, for example, all had the same initial contrasts and step sizes. The approximate step sizes for each experimental color were ±0.002 mechanism contrast for reddish and greenish, ±0.013 mechanism contrast for bluish and yellowish, and ±0.007 for dark gray. Initial contrasts displayed at the beginning of each trial were 0.040 for reddish and greenish, 0.34 for bluish and yellowish, and 0.16 for dark gray.

Each adjustment set contained 100 trials: 20 trials for each of the five experimental colors. Participants completed the adjustment set for one spatial pattern (e.g., gratings) and then continued to the next spatial pattern (e.g., Gaussians). The order of the sets was randomized for different spatial patterns. This was to prevent any effect of the order of spatial pattern.

##### Two-alternative forced-choice

**Figure 2.**
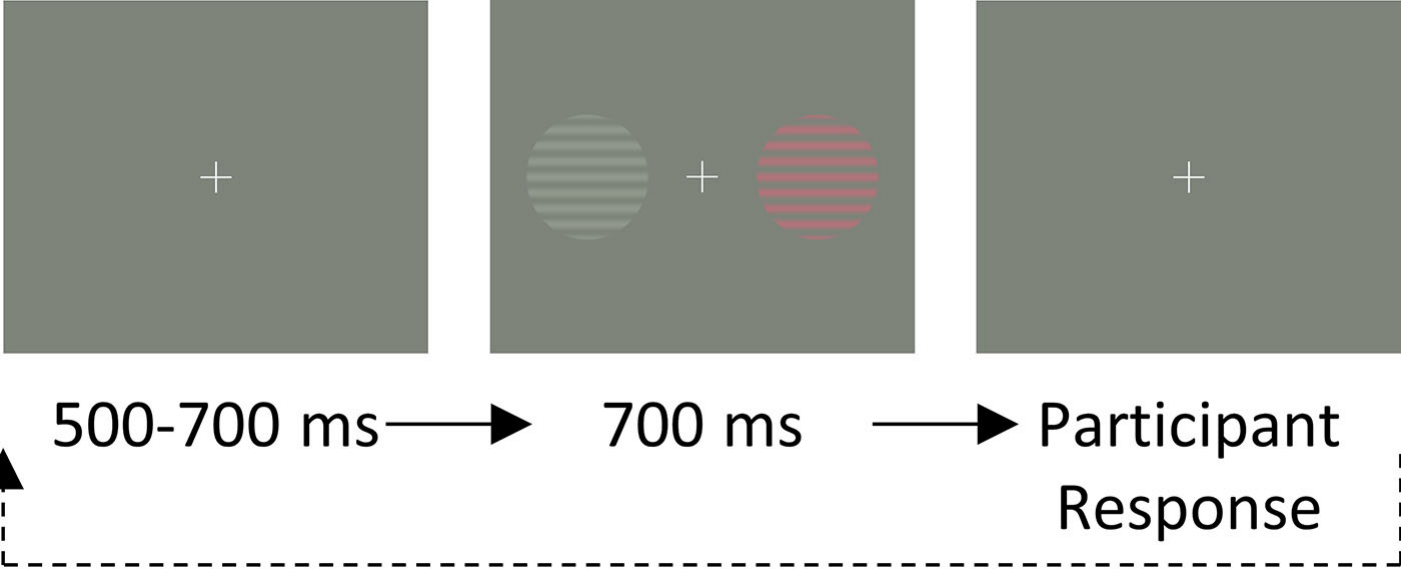
Flowchart of the two-alternative forced-choice task used in Experiment 1. A fixation cross was displayed for 500-700 ms. The stimulus (in this example the “reddish grating” condition) was then displayed for 700 ms. The display then returned to a fixation cross, which was shown until the participant gave a response.

Fifteen participants took part in the 2AFC tasks. Participants were presented with 500 to 700 ms of a fixation cross, followed by 700 ms of the stimulus. The display then returned to the fixation cross until participant response (see Figure 2 for a flowchart of the task). The four-Gaussian condition was not used for this task. Therefore all stimuli had one experimental color and one light gray standard left and right of the fixation. Participants were asked to indicate (via left or right button press) which of the two patches had higher contrast.

The 2AFC used a 1 up/1 down staircase procedure with a termination criterion of 20 reversals. In other words, when participants indicated the standard had higher contrast, the contrast of the experimental color was increased (by a set step size) and when participants indicated the experimental color had higher contrast, its contrast decreased (by the same set step size). A “reversal” is defined as a switch from one direction to another (i.e., changing from “standard has higher contrast” to “experimental color has higher contrast” and vice versa). The staircases were interleaved. The 2AFC continued until the criterion had been met for all five experimental colors.

As with the contrast adjustment task, the different spatial patterns (gratings, Gabors, and Gaussians) were tested separately, with participants completing the five interleaved staircases for one spatial pattern (a “2AFC set”) before continuing onto the next. Also, as up/down adaptive staircases may be biased depending on whether they begin above or below the convergence point, each 2AFC set was run twice for each spatial pattern: one 2AFC began above and one began below the convergence point (point of equal perceived contrast) established through piloting. The average of the results from these two variations was used in any statistical tests performed. As mentioned in the previous section, the order of the sets was randomized for different spatial patterns and initial contrasts.

The initial contrasts varied between spatial patterns (see Table 1 for all values). These initial contrasts were based on pilot data from two experienced observers (the authors, A.H. and J.M.). As with the contrast adjustment task, the step sizes of the 2AFC were constant over the three spatial patterns: ±0.0034 mechanism contrast for reddish and greenish, ±0.020 for bluish and yellowish, and ±0.009 for dark gray.

**Table 1.** Initial contrasts of the above- and below-convergence variations of the 2AFC 1 up/1 down staircases for Experiment 1. *Notes*: The contrasts were equal between reddish (R) and greenish (G) and between bluish (B) and yellowish (Y) conditions but varied among the three spatial patterns (gratings, Gabors, and Gaussians). All values are given in terms of their appropriate mechanism contrast.

Stimulus	Grating			Gabor			Gaussian		
Color	R/G	B/Y	DG	R/G	B/Y	DG	R/G	B/Y	DG
Initial above-convergence contrast (mechanism contrast)	0.068	0.51	0.25	0.068	0.51	0.18	0.027	0.29	0.18
Initial below-convergence contrast (mechanism contrast)	0.034	0.35	0.09	0.034	0.35	0.09	0.008	0.10	0.09

The range of contrast levels along which the staircases could vary was same for all three spatial patterns: 0.001-0.075 for reddish/greenish, 0.010-0.51 for bluish/yellowish, and 0.005-0.30 for dark gray. All values are given in terms of their appropriate mechanism contrast.

##### Detection thresholds

Participants who performed the 2AFC sets also took part in additional tasks that determined their detection thresholds (DTs) for each spatial pattern. The tasks followed the same procedure and used the same three spatial patterns as the 2AFC; however, rather than two patches, only one patch was presented (left or right of the fixation). The patch could be any of the five experimental colors or light gray. Participants were asked to indicate (via left and right button press on the button box) which side of the screen the patch appeared.

Each DT used an adaptive staircase procedure fitted with a Weibull psychometric function (slope of 4; guess-rate of 0.5; lapse rate of 0.02), with a termination criterion of 14 reversals. Staircases for all the stimulus colors were interleaved together pattern by pattern (i.e., all gratings together). As with the adjustment and 2AFC tasks, the detection threshold sets were randomized across spatial patterns.

##### Testing procedure

As adjustment, 2AFC and DT sets were performed for each spatial pattern; there were 13 sets total: four adjustment sets (grating, Gabor, Gaussian and four-Gaussian), three above-convergence 2AFC sets (grating, Gabor and Gaussian), three below-convergence 2AFC sets (grating, Gabor and Gaussian), and three DT sets (grating, Gabor and Gaussian). The order of the sets was randomized. The experiment was performed in multiple sessions lasting up to an hour and a half (including breaks). Most participants completed the experiment in five and a half sessions; the exact timing depended on the speed at which they performed the adjustment sets and how variable they were in the 2AFC and DT sets.

#### Analysis

As a control measure, we analyzed whether the number of Gaussian patches affected the final adjustment score. Comparisons were performed between Gaussian and four-Gaussian results for both reddish/greenish and bluish/yellowish using two two-way repeated measures analyses of variance (ANOVAs), with factors of stimulus number (two and four Gaussians) and color polarity (reddish versus greenish and bluish versus yellowish). The dark gray Gaussian and four-Gaussian results were compared to each other using a paired *t*-test and compared to the light gray standard using one-sample *t*-tests.

To analyze the effects of task, spatial pattern and color, two three-way repeated-measures ANOVAs, with factors of task (adjustment and 2AFC), spatial pattern (grating, Gabor, and Gaussian) and color polarity (reddish vs. greenish and bluish vs. yellowish), were performed on the reddish/greenish and bluish/yellowish data. Significant effects were further examined using Bonferroni corrected paired-samples *t*-tests. The dark gray results were compared using a two-way repeated-measures ANOVA with factors of task (adjustment and 2AFC) and spatial pattern (grating, Gabor and Gaussian). The dark gray results were also compared to the light gray standard using one-sample *t*-tests.

As perceived contrast is dependent on DTs (Kulikowski, 1976), the results from the adjustment and 2AFC tasks were DT-corrected by subtracting the DTs from the adjustment and 2AFC results (Perceived contrast = Physical contrast − Detection threshold).

The reddish/greenish, bluish/yellowish, and dark gray DTs and DT-corrected results were analyzed using the same methods as the original analysis. Because the DTs from the light gray standard were collected for each spatial pattern, the DTs for dark gray and light gray could be compared using a two-way ANOVA with factors of spatial pattern (grating, Gabor, and Gaussian) and luminance polarity (light gray/dark gray). Because the original light gray standard was constant, the “DT-corrected” light gray results were equivalent to the DT results and were therefore not analyzed.

### Results

**Figure 3.**
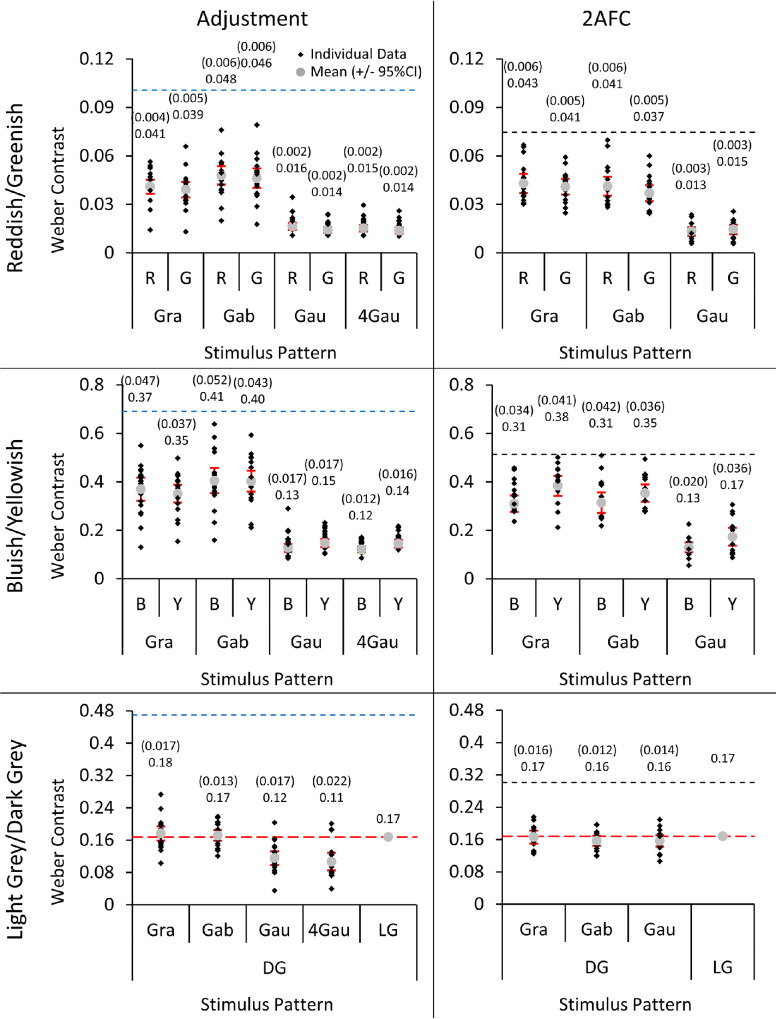
Results of the adjustment (left) and 2AFC (right) tasks for Experiment 1. Reddish/greenish results are on top, bluish/yellowish results are in the middle and light gray/dark gray results are on the bottom. The four spatial patterns are abbreviated as “Gra,” “Gab,” “Gau,” and “4Gau” for “grating,” “Gabor,” “Gaussian,” and “four-Gaussian,” respectively. Individual averages are displayed as black diamonds and overall averages as gray circles. The monitor gamut limits (left) and maximum designated contrasts for the 2AFC staircases (right) are shown as blue and black horizontal dashed lines, respectively. The red dashed lines in the light gray/dark gray graphs correspond to the contrast of the light gray standard. The overall averages and margins of error (1.96*SE; in brackets) are above each data point. The results are shown in terms of their appropriate colour mechanism contrasts. Because light gray was used as the standard, these values are constant for the four spatial patterns and do not have margins of error.

Several participants’ conditions did not converge when performing the 2AFC. This resulted in 14, eight, and 14 participants with data from both staircases for the reddish/greenish, bluish/yellowish, and light gray/dark gray ANOVAs involving task comparisons. This lack of convergence was due to the upper contrast limits set for the 2AFC. Because there were no significant differences between the staircases that started from a lower or a higher level of contrast (all *p*s > 0.085), when data for only one of the two staircases were present, we used the available staircase in place of the average. In the adjustment task, one participant did not perform the four-Gaussian condition and another participant was outside the monitor gamut for bluish gratings. This resulted in comparisons of bluish/yellowish results between Gaussian and four-Gaussian conditions and between grating, Gabor, and Gaussian conditions both having 18 rather than 19 participants with full data. The results of the adjustment and 2AFC tasks are shown in Figure 3.

#### Two versus four gaussians

Two 2 × 2 ANOVAs, with factors of stimulus number (Gaussian and four-Gaussian) and color polarity (reddish and greenish; bluish and yellowish) were performed on the reddish/greenish and bluish/yellowish Gaussian and four-Gaussian results. No effect of stimulus configuration was found for either reddish/greenish (*F*(1,17) = 0.64, *p* = 0.43) or bluish/yellowish (*F*(1,17) = 0.28, *p* = 0.60). A paired *t*-test between the dark gray Gaussian and four-Gaussian data showed there was no effect of stimulus configuration for dark gray either (*t*(17) = 1.31, *p* = 0.21). Because there were no appreciable differences between the results of the Gaussians and four-Gaussians, only the Gaussian results will be considered in subsequent analyses.

#### 2AFC versus adjustment

Two 2 × 3 × 2 ANOVAs (with factors of task, spatial pattern, and color polarity) were performed on the reddish/greenish and bluish/yellowish grating, Gabor and Gaussian results. No effect of task was found (reddish/greenish: *F*(1,14) = 1.69, *p* = 0.21; bluish/yellowish: *F*(1,12) = 0.21, *p* = 0.66); however, there were main effects of spatial pattern (reddish/greenish: *F*(1.33,18.6) = 97.0, *p* < 0.001, η_p_^2^ = 0.48; bluish/yellowish: *F*(1.36,16.3) = 84.8, *p* < 0.001, η_p_^2^ = 0.88) and polarity (reddish/greenish: *F*(1,14) = 5.83, *p* = 0.030, η_p_^2^ = 0.29; bluish/yellowish: *F*(1,12) = 4.94, *p* = 0.046, η_p_^2^ = 0.29). There were also interactions between task and spatial pattern for reddish/greenish (*F*(2,28) = 4.56, *p* = 0.019, η_p_^2^ = 0.25), between task and polarity for bluish/yellowish (*F*(1,12) = 8.56, *p* = 0.013, η_p_^2^ = 0.42) and among all three factors for reddish/greenish (*F*(2,28) = 4.53, *p* = 0.020, η_p_^2^ = 0.24). No other interactions were significant (all *p*s > 0.079). For reddish/greenish, post-hoc comparisons between tasks for each of the three spatial patterns (averaged across polarities) showed no significant effect of task for any of the three spatial patterns after the Bonferroni correction (*p* = 0.017) was applied (gratings: *t*(14) = −1.47, *p* = 0.16; Gabors: *t*(14) = 1.76, *p* = 0.10; Gaussians: *t*(14) = 2.52, *p* = 0.024). For bluish/yellowish, post-hoc comparisons between tasks for the two polarities (averaged across spatial patterns) showed no significant effect of task for either bluish (*t*(12) = 1.55, *p* = 0.15) or yellowish (*t*(13) = −0.76, *p* = 0.46). For the reddish/greenish three-way interaction, comparing the task and spatial pattern interaction between reddish and greenish showed that it held for both greenish (*F*(2,28) = 4.23, *p* = 0.025, η_p_^2^ = 0.26) and reddish (*F*(2,28) = 4.79, *p* = 0.016, η_p_^2^ = 0.23). Post-hoc comparisons between tasks for each of the spatial patterns and for both reddish and greenish (Bonferroni correction 0.008) showed a significant difference between tasks for reddish Gaussians (*t*(14) = 3.60, *p* = 0.003). However, no other comparisons reached significance (all *p*s > 0.17), indicating this was not a more general effect.

A 2 × 3 ANOVA (with factors of task and spatial pattern) performed on the dark gray results showed no effect of task (*F*(1,13) = 1.02, *p* = .33), but significant effects of spatial pattern (*F*(2,26) = 6.78, *p* = .004, η_p_^2^ = .34) and an interaction between the two factors (*F*(2,26) = 5.22, *p* = .012, η_p_^2^ = .29). Post-hoc comparisons between tasks for each spatial pattern showed no difference between tasks for any of the three spatial patterns (gratings: *t*(13) = 1.16, *p* = .27; Gabors: *t*(14) = 1.69, *p* = .11; Gaussians: *t*(14) = -2.08, *p* = .057).

Because the post-hoc tests performed on the reddish/greenish, bluish/yellowish, and dark gray data showed no robust effect of task, and more participants and spatial patterns were tested using the adjustment task, these data were reanalyzed using only the adjustment task results. This was to isolate the effects of spatial pattern and polarity.

#### Adjustments of perceived contrast: Spatial pattern and color polarity

Two 3 × 2 ANOVAs (with factors of spatial pattern and color polarity) were performed on the reddish/greenish and bluish/yellowish results. For reddish/greenish, there was a significant effect of spatial pattern (*F*(2,36) = 83.6, *p* < 0.001, η_p_^2^ = 0.82). There was also a significant effect of color polarity (*F*(1,18) = 5.76, *p* = 0.027, η_p_^2^ = 0.24), with greenish requiring significantly less contrast compared to reddish. However, there was no interaction between spatial pattern and color polarity (*F*(2,36) ≤ 0.20, *p* = 0.82). Post-hoc paired-samples *t*-tests between the three spatial patterns (averaged across reddish and greenish; Bonferroni corrected *p* = 0.017) showed Gaussians required significantly less physical contrast to reach equal perceived contrast with the standard compared to gratings (*t*(18) = −9.70, *p* < 0.001) and Gabors (*t*(18) = −10.3, *p* < 0.001) and gratings required significantly less physical contrast compared to Gabors (*t*(18) = −3.74, *p* = 0.002). For bluish/yellowish, there was a significant effect of spatial pattern (*F*(2,34) = 85.4, *p* < 0.001, η_p_^2^ = 0.83), but not color polarity (*F*(1,17) < 0.001, *p* = 0.98). However, there was an interaction between the two factors (*F*(2,34) = 7.30, *p* = 0.002, η_p_^2^ = 0.30). Post-hoc paired-samples *t*-tests among the three spatial patterns (averaged across bluish and yellowish; Bonferroni corrected *p* = 0.017) showed Gaussians required significantly less physical contrast to reach equal perceived contrast with the standard compared to gratings (*t*(17) = −9.51, *p* < 0.001) and Gabors (*t*(18) = −10.4, *p* < 0.001) and gratings required significantly less physical contrast compared to Gabors (*t*(17) = −2.84, *p* = 0.011). Paired-samples *t*-tests between bluish and yellowish for the three spatial patterns (Bonferroni corrected *p* = 0.017) showed bluish required less physical contrast to achieve equal perceived contrast with the standard compared to yellowish for Gaussians (*t*(17) = −3.43, *p* = 0.003), meaning that bluish was more salient than yellowish. No difference was found between bluish and yellowish for gratings (*t*(17) = 2.34, *p* = 0.031) and Gabors (*t*(17) = 0.20, *p* = 0.84).

A one-way ANOVA between the three spatial patterns was performed on the dark gray results. A significant effect was found (*F*(2,36) = 16.7, *p* < 0.001, η_p_^2^ = 0.48). Post hoc paired-samples *t*-tests (Bonferroni corrected *p* = 0.017) showed no differences between gratings and Gabors (*t*(18) = 0.69, *p* = 0.50). However, less physical contrast was required to achieve equal perceived contrast with the standard for Gaussians compared to gratings (*t*(18) = −5.07, *p* < 0.001) and Gabors (*t*(18) = −4.89, *p* < 0.001).

Because dark gray and light gray come from the same color mechanism, the dark gray results were directly compared to the light gray standard using three one-sample *t*-tests. Physical contrast for dark gray was equivalent to the light gray standard contrast for gratings (*t*(18) = 0.98, *p* = 0.34) and Gabors (*t*(18) = 0.59, *p* = 0.56). However, dark gray required less physical contrast than the light gray standard to achieve equal perceived contrast for Gaussians (*t*(18) = −3.54, *p* = 0.002).

#### Detection thresholds and DT-based correction of adjustment data

**Figure 4.**
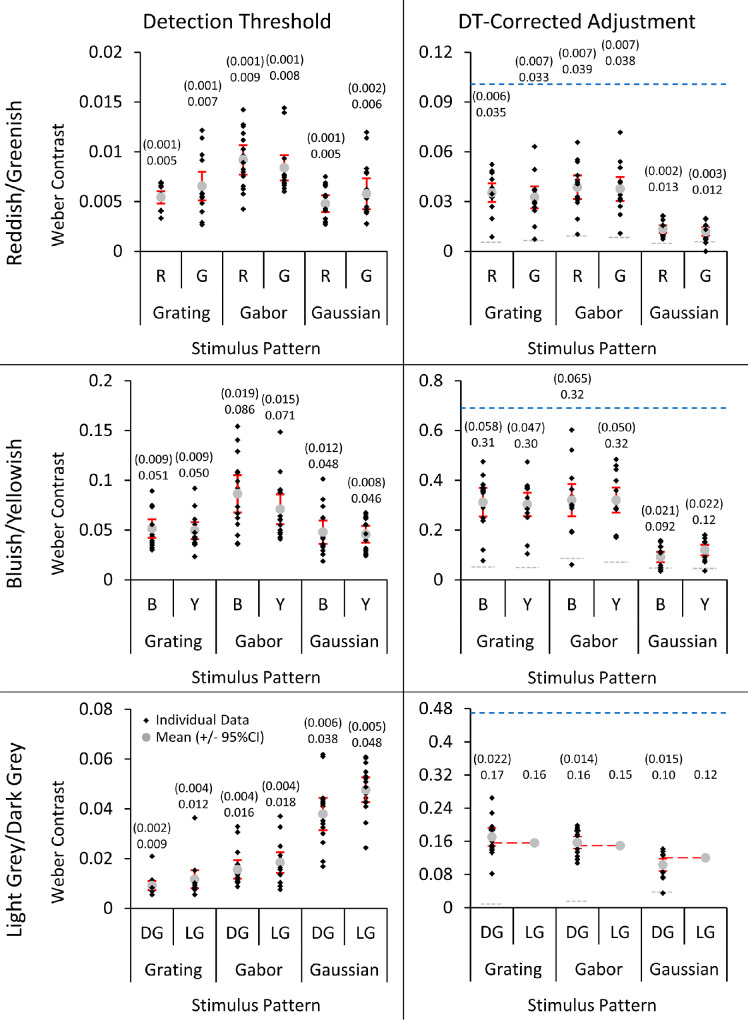
Detection thresholds (DT) (left) and detection threshold–corrected contrasts of the adjustment task (right) for reddish/greenish (top), bluish/yellowish (middle), and light gray/dark gray (bottom) for Experiment 1. Note that the DT and DT-corrected adjustment task contrast graphs have different y-axis scales. Individual averages are displayed as black diamonds and overall averages as gray circles. The monitor gamut limits and average detection thresholds are shown on the DT-adjusted graphs as blue and gray horizontal dashed lines, respectively. The red dashed lines in the light gray/dark gray graph correspond to the DT-adjusted contrasts of the light gray standards for each spatial pattern. The overall averages and margins of error (1.96*SE; in brackets) are above each data point. The results are shown in terms of their appropriate mechanism contrasts.

Perceived contrast is dependent on DTs. Therefore the DTs for each spatial pattern (gratings, Gabors, and Gaussians) were measured and then subtracted from the “physical contrast” results analyzed above. The average DTs and the DT-corrected contrasts for the adjustment task are shown in Figure 4.

To assess how far above detection threshold the contrast matching results were, ratios of contrast match/detection threshold were calculated. On average, contrast matches for gratings were 6.67, 7.13, and 15.7 times detection threshold for reddish/greenish, bluish/yellowish, and dark gray, respectively. For Gabors the ratios were 5.53, 5.16, and 10 times, respectively. Gaussians had the lowest ratios: 2.73, 2.98, and 3.37 times detection threshold, respectively.

Three 3 × 2 ANOVAs (with factors of spatial pattern and color polarity) were performed for the reddish/greenish, bluish/yellowish, and light gray/dark gray DTs. There was a significant effect of spatial pattern for all three color mechanisms (reddish/greenish: *F*(2,28) = 30.0, *p* < 0.001, η_p_^2^ = 0.68; bluish/yellowish: *F*(1.46,20.4) = 24.2, *p* < 0.001, η_p_^2^ = 0.63; light gray/dark gray: *F*(2,28) = 113, *p* < 0.001, η_p_^2^ = 0.89) and a significant effect of color polarity for bluish/yellowish (*F*(1,14) = 5.28, *p* = 0.037, η_p_^2^ = 0.27) and light gray/dark gray (*F*(1,14) = 24.2, *p* < 0.001, η_p_^2^ = 0.63) but not reddish/greenish (*F*(1,14) = 2.81, *p* = 0.12). An interaction between spatial pattern and color polarity was found for all three color mechanisms (reddish/greenish: *F*(2,28) = 3.92, *p* = 0.031, η_p_^2^ = 0.22; bluish/yellowish: *F*(2,28) = 5.01, *p* = 0.014, η_p_^2^ = 0.26; light gray/dark gray: *F*(1.40,19.6) = 11.9, *p* = 0.001, η_p_^2^ = 0.46). Paired-samples *t*-tests (Bonferroni corrected *p* = 0.017) between reddish and greenish showed there were no significant differences between the two color polarities for any of the three spatial patterns (gratings: *t*(14) = −2.15, *p* = 0.050; Gabors: *t*(14) = 1.72, *p* = 0.11; Gaussians: *t*(14) = −1.80, *p* = 0.093). Paired-samples *t*-tests between bluish and yellowish showed DTs were significantly higher for bluish compared to yellowish for Gabors (*t*(14) = 3.11, *p* = −0.008), but not significantly different for gratings (*t*(14) = 0.84, *p* = 0.41) and Gaussians (*t*(14) = 0.52, *p* = 0.61). Paired-samples *t*-tests between light gray and dark gray showed DTs were significantly higher for light gray compared to dark gray for Gabors (*t*(14) = 3.63, *p* = 0.003) and Gaussians (*t*(14) = 4.69, *p* < 0.001), but not significantly different for gratings (*t*(14) = 2.50, *p* = 0.026).

Two 3 × 2 ANOVAs (with factors of spatial pattern and color polarity) were also performed for the reddish/greenish and bluish/yellowish DT-corrected adjustment task results. Both had a significant effect of spatial pattern (reddish/greenish: *F*(1.36,16.3) = 40.5, *p* = 0.031, η_p_^2^ = 0.77; bluish/yellowish: *F*(2,26) = 52.5, *p* < 0.001, η_p_^2^ = 0.80), but no significant effect of color polarity (reddish/greenish: *F*(1,12) = 2.56, *p* = 0.14; bluish/yellowish: *F*(1,13) = 1.07, *p* = 0.32). Although there was no significant interaction for reddish/greenish (*F*(2,24) = 0.48, *p* = 0.62), an interaction was found between spatial pattern and color polarity for bluish/yellowish (*F*(2,26) = 6.78, *p* = 0.004, η_p_^2^ = 0.34). For reddish/greenish, paired-samples *t*-tests among the three spatial patterns (averaged across color polarities; Bonferroni corrected *p* = 0.017) showed that, even when corrected using DTs, Gaussians required significantly lower physical contrast to achieve equal perceived contrast with the standard compared to gratings (*t*(12) = −7.91, *p* < 0.001) and Gabors (*t*(12) = −6.44, *p* < 0.001), which were not significantly different from each other (*t*(14) = −2.07, *p* = 0.058). Likewise, bluish/yellowish paired *t*-tests among the three spatial patterns (averaged across color polarities; Bonferroni corrected *p* = 0.017) showed Gaussians required significantly lower physical contrast to achieve equal perceived contrast with the standard compared to gratings (*t*(13) = −7.93, *p* < 0.001) and Gabors (*t*(14) = −7.72, *p* < 0.001), which were not significantly different from each other (*t*(13) = −0.26, *p* = 0.80). Paired-samples *t*-tests (Bonferroni corrected *p* = 0.017) between bluish and yellowish showed that, when corrected using DTs, there were no significant differences between bluish and yellowish for gratings (*t*(13) = 1.93, *p* = 0.076) and Gabors (*t*(14) = −0.023, *p* = 0.98); however, bluish Gaussians were perceived as having higher contrast than yellowish (required less physical contrast to achieve equal perceived contrast with the standard; *t*(13) = −4.92, *p* < 0.001).

A one-way ANOVA between the spatial patterns was performed on the dark gray DT-corrected adjustment task results (*F*(2,28) = 41.7, *p* < 0.001, η_p_^2^ = .75). Post hoc paired-samples *t*-tests (Bonferroni corrected *p* = 0.017) showed that, when corrected using DTs, there are no differences between gratings and Gabors (*t*(14) = 1.65, *p* = 0.12). However, Gaussians still required less contrast to achieve equal perceived contrast with the standard compared to gratings (*t*(14) = −8.22, *p* < 0.001) and Gabors (*t*(14) = −7.47, *p* < 0.001). Three one-sample *t*-tests (Bonferroni corrected *p* = 0.017) comparing the DT-corrected dark gray results to the DT-corrected light gray standards for each spatial pattern (light gray standard contrast minus the average DT of each spatial pattern) showed no significant differences between the DT-corrected dark gray results and light gray standards (grating: *t*(14) = 1.27, *p* = 0.22; Gabor: *t*(14) = 1.05, *p* = 0.31; Gaussian: *t*(14) = −2.26, *p* = 0.040).

### Interim discussion

Reddish, greenish, bluish, yellowish, and dark gray were matched for perceived contrast with light gray for different spatial patterns. Detection thresholds (DTs) were also measured and subtracted from the matching results to correct for any perceived contrast asymmetries caused by asymmetries in DTs, as suggested by Kulikowski (1971). Neither task (2AFC or adjustment) nor number of elements (two or four Gaussians) affected the contrast matching results. Gaussians required significantly less physical contrast to achieve equal perceived contrast with the standard compared to either gratings or Gabors. Because Gaussians are lowpass patterns, it is likely that they preferentially activate single-opponent, edge-insensitive cells, which are more prominent in color vision. This is supported by the finding that while thresholds for chromatic Gaussians were lower than those for Gabors, the opposite pattern was found for luminance-defined stimuli, in line with what could be predicted from CSFs (Mullen, 1985). Consistent with these threshold differences, we also observe the following asymmetry between color and luminance for contrast matches to a light gray standard: whereas for dark gray, perceived contrast after DT subtraction is equated for the three patches, for chromatic stimuli it still remains ∼2 to 3.5 times lower for Gaussians compared to gratings and Gabors.

Dark gray Gaussians achieved equal perceived contrast at a lower contrast than that contained within the light gray standard. The higher sensitivity and salience for darker stimuli is also consistent with previous neurophysiological and psychophysical findings (Komban, Alonso, & Zaidi, 2011; Xing, Yeh, & Shapley, 2010) and also explains why, after DT-correction, dark gray Gaussians’ contrast was no longer significantly different from the DT-corrected light gray standard. Finally, bluish required less physical contrast to achieve equal perceived contrast with the standard compared to yellowish only for Gaussians. These Gaussian-specific effects replicate the findings of Hardman et al. (2020), which observed the same effect using contrast matching with a stimulus that consisted of eight small Gaussians. The differences between bluish and yellowish Gaussians persisted even after adjustment using DTs. In other words, bluish Gaussians were perceived as having higher contrast than yellowish Gaussians when at equal levels of physical contrast, and this difference remained even after subtraction of DTs. The lack of asymmetry for gratings may, in fact, be due to their contrast matches being higher above their detection thresholds compared to Gaussians (∼7 times above, compared to ∼3 for Gaussians). In comparison, contrast constancy for orientation discrimination emerges at ∼3 times above DT (Regan & Beverley, 1985). A future study should assess more fully the contrast dependency of the asymmetries in perceived contrast for gratings reported by Switkes (2008).

This experiment did not include contrast matches between different spatial patterns. Thus direct comparisons of the perceived contrasts of the three spatial patterns cannot be made. Figure 3 shows that whereas most participants’ datapoints fall near the mean, there is a small number of participants that produce either much smaller or much larger contrast matches. Hurvich and Jamieson (1954) reported that variability in heterochromatic brightness matching increases with increasing wavelength separation between the standard and the comparison stimulus. We observe that larger variability for color-to-luminance matching as opposed to luminance-to-luminance matching is much more pronounced for gratings and Gabors. For these two spatial patterns, comparisons between the 1 cpd periodic structure would rely on different CSF envelopes for color and luminance. In fact, Georgeson (1991) discusses how the most viable model of contrast encoding incorporates the dependence of contrast matching on threshold (P = C – T) but that this simple dependence holds only if differences in gain between different channels are compensated. Thus the subtractive formula for perceived contrast suggested by Kulikowski (1976) may not be able to correct results for matches between chromatic and achromatic stimuli.

## Experiment 2 –perceived contrast of lowpass stimulus patterns

The unique spatial properties of the Gaussian cause it to have a generalized difference in contrast matches compared to gratings and Gabors *and* a specific asymmetry in perceived contrast between bluish and yellowish (bluish requiring less perceived contrast than yellowish to achieve equal perceived contrast with a standard, as observed earlier by Hardman et al., 2020). Gaussians more strongly activate single-opponent neurons and lowest spatial frequency channels while gratings and Gabors more strongly activate higher spatial frequency channels and double-opponent neurons. Because the bluish/yellowish asymmetry in perceived contrast matches was present in Gaussians but not gratings or Gabors, it may be related to this same matter. Alterations in the lowpass internal modulation of contrast could affect the differential excitation of S + and S − mechanisms (e.g., Tailby et al., 2008). Another, related explanation could be that the appearance of an external edge in uniform patches leads to a stronger activation of double-opponent cells (Nunez et al., 2018). Although double-opponent neurons contain cone-opponent inputs, as occur in single-opponent V1 neurons, they also have spatially opponent inputs (Shapley & Hawken, 2011). This means that they have areas within their receptive field excited by one type of cone activity and other areas within their receptive field *inhibited* by *the same* cone activity. In terms of the S + /S − asymmetry, this would mean higher activity for S + neurons compared to S − neurons would be cancelled out for double-opponent V1 neurons, because, although there would be areas of greater excitation for S + compared to S −, there would also be areas of greater *inhibition* for S + compared to S − . Thus bluish/yellowish asymmetry would not be present for V1 double-opponent neurons, resulting in no asymmetry in perceived contrast once they begin to make a significant contribution to contrast processing.

This experiment will test these hypotheses by varying (1) the average contrast per area and (2) spatial distribution of contrast. This will be achieved by creating patches of equal size and maximum contrast that contain a central uniform-contrast area of varying size surrounded by an area of linearly decreasing contrast. Because higher average contrast per area is bound to result in higher perceived contrast of the standard, participants should require increasing contrast to achieve equal perceived contrast with it. In other words, the perceived contrast of the standard will increase with increasing size of the central uniform-contrast area (and therefore increasing average contrast per area). Furthermore, we predict that the two colors’ perceived contrast asymmetry should decrease as the patch moves from a largely non-flat, lowpass contrast distribution towards a fully flat, uniform contrast patch. If this transition is mostly driven by LSF content there should be a gradual reduction in perceived contrast asymmetry. On the contrary, a major contribution from double-opponent cells would lead to a more step-wise change, with the asymmetry being present for all the non-flat stimuli but being eliminated for the uniform patch.

### Materials and methods

#### Participants

This experiment tested twelve participants (eleven female, all right-handed, age range 20–25; *M* = 21). All participants had normal or corrected-to-normal vision and no history of neurological or psychiatric disorders.

#### Stimuli

Because an asymmetry was seen only in the bluish/yellowish Gaussians of the previous experiment when results were corrected using DTs, only the bluish and yellowish cardinal DKL axis colors were used as experimental colors. In the previous experiment, some participants wished to increase the contrast beyond the range of the monitor gamut—and this happened mainly for bluish/yellowish. To prevent the limits of the monitor gamut from interfering with the measurements, the light gray standard used a somewhat lower contrast (Weber contrast of 0.12; 0.05 lower than that of Experiment 1, with CIE 1931 coordinates of 0.3002, 0.3125, 52.10 cd/m^2^). However, this contrast was still higher than that of Switkes (2008) by 0.04 rms contrast (0.21 compared to 0.17).

Four different patches were created that contained an area of uniform contrast (“center”) surrounded by an area of linearly decreasing contrast (the “surround”; beginning at the contrast of the uniform area and decreasing to zero contrast, equivalent to the neutral background, in twenty steps of contrast).

The patches varied in the radius of the center (i.e. their level of internal contrast modulation). The first had a “center” radius of 40% of the total patch (i.e., because the patch had a radius of 5° visual angle, the “center” had a radius of 2° visual angle). The other three had increasing radii for the center (60%, 80%, and 100% of total patch). In the case of the 100% patch, the “center” covered the entire patch, resulting in a circle of uniform contrast, with a hard external edge.

**Figure 5.**
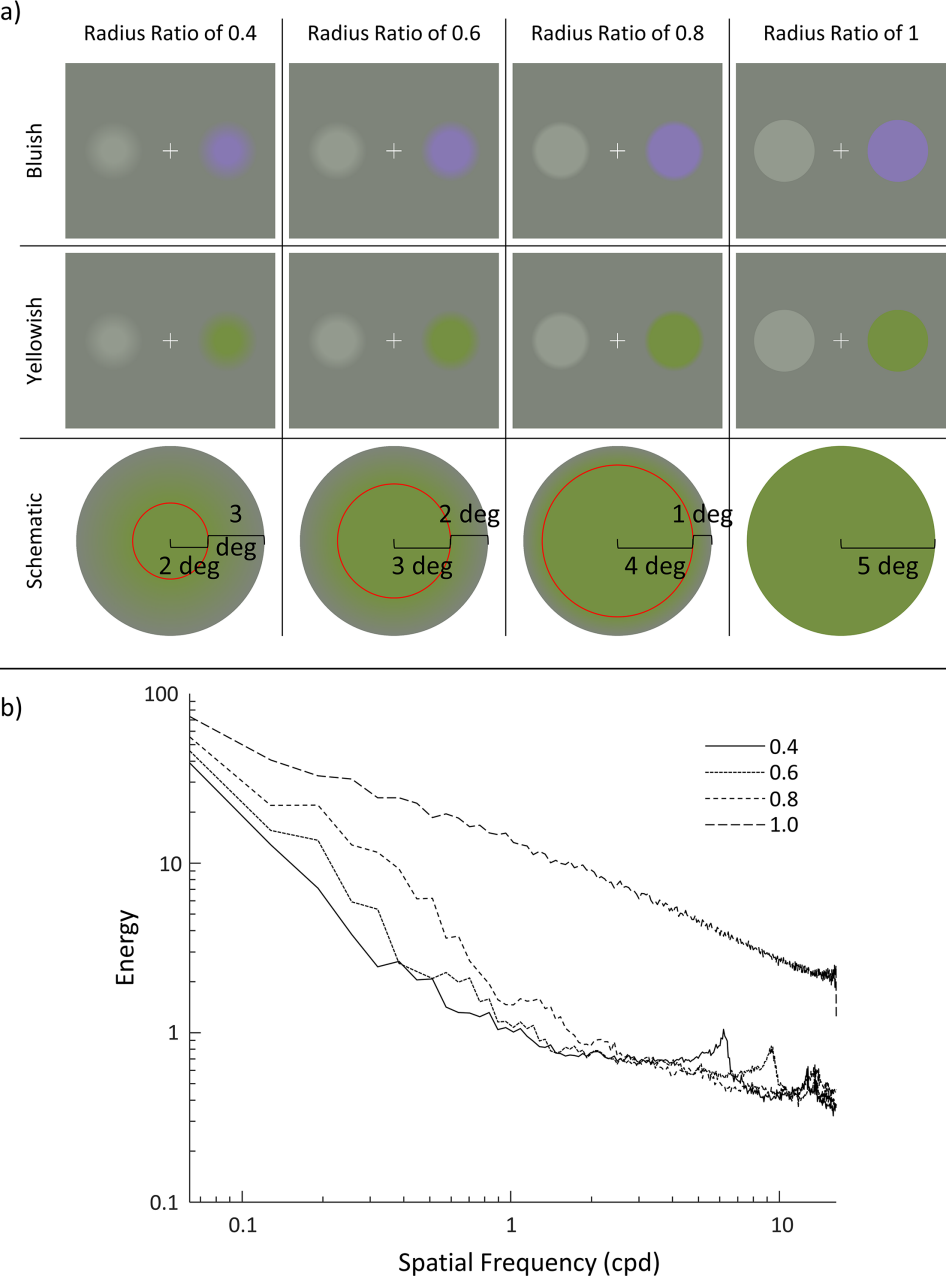
Examples of the stimuli used in Experiment 2 are shown in panel (a). The columns show the four center/total radius ratios (0.4, 0.6, 0.8 and 1). The rows show the two colors (bluish and yellowish) and a schematic representation of the center and surround components of each center/total radius ratio. The red line present in the schematic representations is for illustration purposes only and was not present in the actual experiment. Panel (b) shows the contrast magnitude of four center/total radius ratio conditions plotted as a function of their spatial frequency. Note the graph is plotted on a log-log scale.

The overall visual display consisted of two patches (varying only in color), left and right of the fixation cross: one was the light gray standard, the other was one of the two experimental colors. Conditions will be referred to by their color (B and Y) and the ratio of “center” radius to total radius (0.4, 0.6, 0.8 and 1): B_0.4_, B_0.6_, B_0.8_, B_1_, Y_0.4_, Y_0.6_, Y_0.8_, and Y_1_. Examples of the conditions and a schematic representation of their “center” and “surround” components are shown in Figure 5a. As with Experiment 1, to enable comparison of stimulus contrast energy and spatial frequency content, the power spectra were plotted as a function of their spatial frequency in Figure 5b.

#### Heterochromatic flicker photometry

HCFP was used to measure the point of isoluminance for bluish and yellowish. The step size was ±0.008 Weber luminance contrast and the degree of elevation displayed at the beginning of each trial was randomized (trial-by-trial) within the range of 0 ± 0.041. Because there were four center/total radius ratios (0.4, 0.6, 0.8, and 1), the HFCP procedure was performed for each spatial pattern separately.

#### Task

Participants performed the contrast adjustment procedure (outlined in General Methods) for each of the four center/total radius ratios (and both colors). The approximate step size was ±0.013 mechanism contrast and the initial contrast displayed at the beginning of each trial was 0.34 for bluish and yellowish. All conditions were run together in a randomized order; because there were eight total conditions and 20 trials per condition, the experiment contained 160 trials.

#### Analysis

To compare the results of center/total radius ratio and color, a two-way repeated-measures ANOVA, with factors of radius ratio (0.4, 0.6, 0.8, and 1) and color (bluish and yellowish) were performed. Significant effects were further examined using Bonferroni corrected paired-samples *t*-tests.

A principal component analysis was performed to identify whether differences between the center/total radius ratios and between the colors were largely due to a single overarching factor or whether different mechanisms were responsible for either different center/total radius ratios or different colors. Factor analysis is a suitable statistical approach to infer the presence of latent factors that can account for the shared variance in a set of observed variables (Peterzell, 2016). We did not rotate the solution because we did not want to impose either dependence or independence of the observed latent factors. A criterion of Eigen values greater than one was used to determine the number of factors to retain. A brief but informative explanation for the benefits of confirmatory and exploratory factor analysis, particularly for experiments with stimuli that vary along continuous dimensions such as contrast, spatial frequency and hue angle, may be found in the “Factor Analytic Approaches” section of Mollon, Bosten, Peterzell, and Webster's (2017) review of examining individual differences in vision science.

### Results

**Figure 6.**
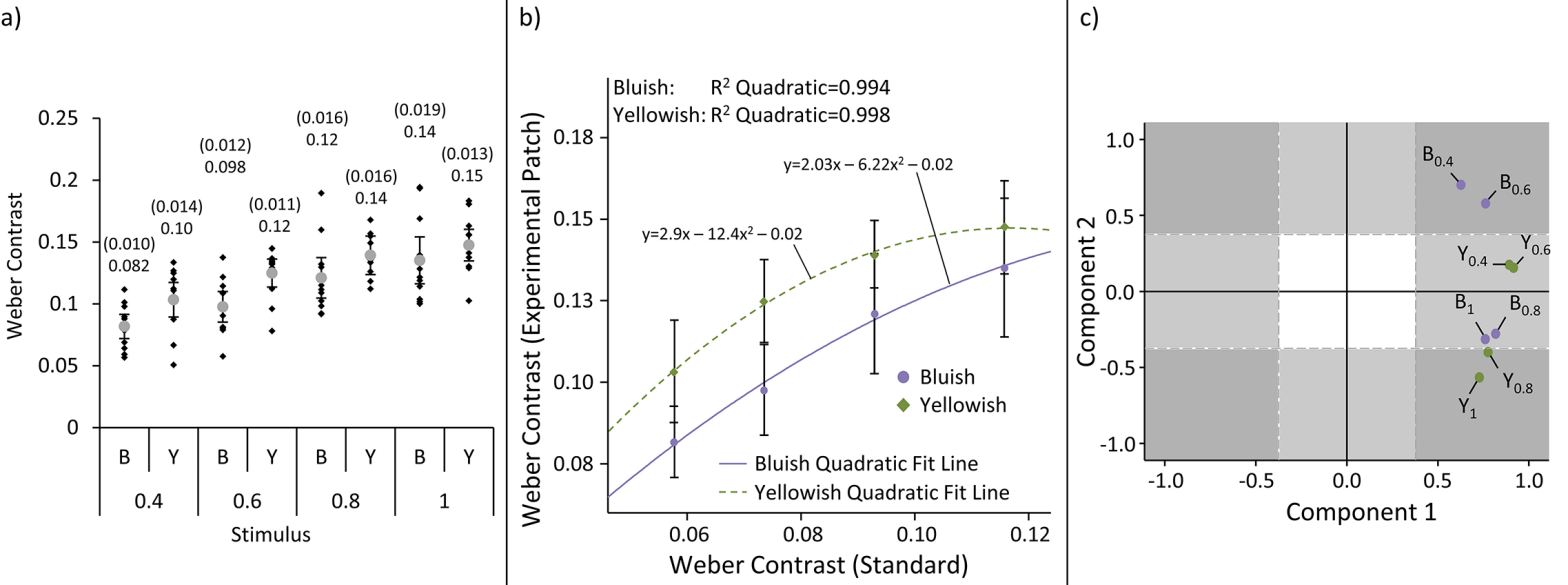
Results of Experiment 2. The four center/total radius ratios are abbreviated as 0.4, 0.6, 0.8, and 1. Panel (a) shows the contrast adjustment results of the eight conditions. Individual averages are displayed as black diamonds and overall averages as gray circles. The overall averages and 1.96*SE (in brackets) are above each section of the data. The results are shown in terms of bluish/yellowish color mechanism contrast. Panel (b) shows the average contrasts of the experimental patch (y axis) versus the standard patch contrast (x axis). Bluish and yellowish are each depicted with their own color. The quadratic fit lines, their equations and *R*^2^ values are also displayed. Panel (c) shows the component plot of the principal component analysis. The factor loadings are listed in Table 2. Dotted lines show the ±0.4 factor loading lines designating significant values. The shaded areas show the areas where either one (light gray) or both (dark gray) components would have significant factor loadings.

All results from this experiment are shown in Figure 6 and Table 2. In the first experiment, mean S − (L + M) contrast at match was 0.36 for gratings, 0.405 for Gabors and 0.14 for Gaussians. In Experiment 2, the contrast of the light gray standard was 29% lower than in Experiment 1. To assess whether our results between experiments are consistent, we compared contrast matches for Gaussians in Experiment 1 with those for 0.4 and 0.6 radius ratio stimuli in Experiment 2, because they are the most Gaussian-like. Assuming a linear relationship for contrast matching to different light gray standards, average matches of 0.091 for 0.4 radius ratio and 0.109 for 0.6 radius ratio to a standard of 0.12 fit relatively well with the predicted match for a Gaussian (0.14 – 0.0406 = 0.0994; − 0.0406 is subtracted because it is 29% of the matched contrast in Experiment 1).

**Table 2.** Factor loadings, Eigen values and explained variance for Components 1 and 2 of the principal component analysis for Experiment 2.

Stimulus	Component 1	Component 2
Y_0.4_	0.92	0.16
B_0.4_	0.63	0.71
Y_0.6_	0.89	0.18
B_0.6_	0.76	0.58
Y_0.8_	0.78	−0.40
B_0.8_	0.82	−0.28
Y_1_	0.73	−0.57
B_1_	0.76	−0.31
Eigen value	4.99	1.55
Explained variance	62.3%	19.3%
Total explained variance		81.7%

#### Comparing across color and center/total radius ratio

Results are shown in Figure 6a. A 4 × 2 ANOVA (with factors of center/total radius ratio and color polarity) showed there was a significant effect of radius ratio (*F*(1.62,17.8) = 33.0, *p* < 0.001, η_p_^2^=.75), with paired-samples *t*-tests (Bonferroni corrected *p* = 0.008) showing 0.4 center/total radius ratio conditions required significantly lower physical contrast to achieve equal perceived contrast with the standard compared to all other conditions (0.6 ratio: *t*(11) = −6.25, *p* < 0.001; 0.8 ratio: *t*(11) = −6.82, *p* < 0.001;1 ratio: *t*(11) = −7.43, *p* < 0.001). Likewise, the 0.6 ratio conditions required significantly lower physical contrast to achieve equal perceived contrast with the standard compared to the 0.8 ratio (*t*(11) = −3.78, *p* = 0.003) and 1 ratio (*t*(11) = −4.42, *p* = 0.001) conditions. The 0.8 and 1 ratio conditions were not significantly different from each other (*t*(11) = −3.02, *p* = 0.012). A significant effect of color polarity was also found (*F*(1,11) = 19.9, *p* = 0.001, η_p_^2^ = 0.64), with yellowish requiring higher physical contrast to achieve equal perceived contrast with the standard compared to bluish. Although no linear interaction was found (*F*(1.36,14.9) = 2.05, *p* = 0.17), there was a quadratic interaction between color polarity and radius ratio (*F*(1.36, 14.9) = 5.06, *p* = 0.046, η_p_^2^ = 0.32). In other words, theoretical regression equations for the two color polarities would be [contrast_bluish_ = *a*_bluish_(radius ratio) + *b*_bluish_(radius ratio)^2^ + *c*] and [contrast_yellowish_ = *a*_yellowish_(radius ratio) + *b*_yellowish_(radius ratio)^2^ + *d*], where *a*_bluish_ and *b*_bluish_ and *a*_yellowish_ and *b*_yellowish_ are the regression coefficients for the bluish and yellowish equations and *c* and *d* are constants representing the bluish and yellowish regression intercepts. This quadratic relationship is possible because the “radius ratio” variable can be treated as continuous: the average contrasts for each standard patch can be used as the independent variable in the two theoretical equations above. Figure 6 b shows a plot of the average experimental contrasts versus the average contrasts of the standard patch, as well as quadratic fit lines for bluish and yellowish results. Because both experimental and standard patches had identical sizes and contrast distributions, this is equivalent to comparing the average contrast per area as well.

#### Factor analysis

Differences in the quadratic relationship between bluish and yellowish suggest that a general contrast metric (e.g., average contrast per area) is not sufficient to explain our findings and that at least one additional factor is required. To determine these underlying factors, a principal component analysis was performed. The component plot of the analysis is shown in Figure 6c. Table 2 lists the component loadings for each component, their Eigen values and the variance explained by the components separately and cumulatively. As stated in Pituch and Stevens (2016), factor loadings >0.4 were deemed to be significant, yielding a minimum *r*^2^ of 0.16.

Two components had Eigen values above 1, which cumulatively explain 81.7% of the variance. The first is an overall factor that loads heavily for all conditions and explains 62.3% of the variance, with component loadings ranging from 0.63 to 0.92. This component is probably related to the average contrast per area of the standard, which would be the main factor determining the comparison setting. A second component loads positively for 0.4 and 0.6 center/total radius ratio conditions and negatively for 0.8 and 1 center/total radius ratio conditions. It explains 19.3% of the variance, with positive component loadings ranging from 0.18 to 0.71 and negative component loadings ranging from −0.28 to −0.57. The component loadings with an absolute value of 0.4 or above are taken to be large enough to make a meaningful contribution as they explain at least 16% of variance: here, these significant contributors are positive loadings for bluish at 0.4 and 0.6 radius ratio and negative loadings for yellowish at 0.8 and 1 radius ratio. Therefore the second factor relates to the differences between bluish and yellowish perceived contrast because of the spatial structure of the patch, with a dissociation between conditions with a uniform center over more than half of their area (0.8 and 1 center/total radius ratio conditions) and less than half of their area (0.4 and 0.6 center/total radius ratio conditions).

## General discussion

Although many studies observe asymmetries in detection thresholds, discrimination thresholds, and perceived contrast of various colors, to our knowledge this experiment is the first to determine whether changes to the stimulus spatial pattern or experimental task affect these asymmetries in a relatively large sample of participants. This was achieved by measuring perceived contrast matches of various colors to a light gray standard for several combinations of spatial patterns. The first experiment measured perceived contrast matches for all cardinal colors in a physiological, cone-opponent color space using three different spatial patterns. Gaussians produced lower contrast matches compared to gratings and Gabors for color but not luminance-defined stimuli. Furthermore, Gaussians were also unique in that they alone produced an asymmetry in perceived contrast matches between bluish/yellowish. The second experiment expanded on these results by focusing on which properties of the lowpass stimulus produced the specific bluish/yellowish contrast match asymmetry. Shifting the presented stimulus from a lowpass spatial pattern similar to a Gaussian into a uniform circular patch both increased the overall contrast match and reduced the asymmetry between bluish and yellowish contrast matches, demonstrating that different low spatial frequency carriers are driving perceived contrast for S+ and S- colors. Our findings do not only contribute to our understanding of low-level physiological mechanisms but also have serious implications for studies that aim to compare color and luminance mechanisms, for example, in terms of choosing spatiochromatic properties of stimuli for neuroimaging studies, because appearance of luminance and chromatic patches may be noticeably different because of the underlying low-level channels that provide the major inputs into perceived contrast.

Contrast matches increased with increasing average contrast per area. Furthermore, in our second experiment, principal component analysis revealed that the main factor that explained 62% of contrast matching performance loaded strongly and positively for all conditions, suggesting that it probably reflected a general measure of stimulus contrast. Several candidate contrast measures could drive this effect: the non-flat contrast per area, the flat contrast per area and the average contrast per area. The average contrast per area is the most likely of these contrast measures. It increases non-linearly due to the circular shape of the patches, explaining the quadratic nature of the relationship between the standard and experimental patch contrasts in Experiment 2. Furthermore, it incorporates the other contrast measures of center and surround contrast per area, which are necessarily linked to both each other and the average contrast per area due to the nature of the stimulus.

Because multiple parameters necessarily vary in concert within the different patches, a principal component analysis was performed to ascertain which additional factors, besides average contrast per area, could explain the variation in experimental results. Although more than half (62%) of the variance was explained by one overarching factor, a second, weaker factor was found that divided conditions into bluish patches with uniform maximum contrast over less than half of the total patch area and yellowish patches with uniform maximum contrast over more than half of the total patch area. It seems likely that this is related to different neuronal contributions to patches’ perceived contrast: when less than half of the patch is uniform, these neurons’ activity will result in perceived contrast being higher for bluish than yellowish, and when more than half of the patch is uniform, the resultant activity will be in favor of yellowish as opposed to bluish.

Although Experiment 2 varied the average contrast per area using the size of the uniform central patch, the coincidental variation in the lowpass component of the stimulus pattern also altered the preferential activation of spatial frequency detectors. Smaller radius ratio patches had smoother internal contrast modulation, mimicking the spatial pattern of Gaussians and preferentially activating single-opponent and low spatial frequency tuned neurons. Increasing the proportion of the flat contrast area from 40% to 60% led to a small increase in contrast magnitude mainly concentrated around 0.1 to 0.3 cpd whereas a further increase to 80% increased the magnitude across 0.1 to 0.7 cpd (see Figure 5b). Based on the factor analysis, this 80% condition produced similar results to the uniform patch, suggesting that the divide between the 40% and 60% conditions and the 80% and uniform patch conditions was rooted in these differences in contrast magnitude present in the 0.1 to 0.7 cpd range. This also ties in well with Tailby, Solomon, and Lennie's (2008) and Tailby, Szmajda, et al.'s (2008) findings that S+ and S− LGN neurons are preferentially tuned to low spatial frequencies, with their highest firing rates occurring from 0.3 cpd and uniform field stimuli, respectively. Meanwhile, the uniform patch had a contrast energy signature that would activate both single-opponent neurons in the center of the patch and double-opponent neurons toward the outer edge. Thus the results from both experiments are also in line with the hypothesis that lower contrast matches for patches with lowpass non-flat contrast distributions are due to higher responses to color compared to luminance found for low spatial frequency and single-opponent neurons. This interpretation is consistent with neurophysiological findings. Solomon, Lee, White, Rüttiger, and Martin (2005) previously demonstrated that both cone-opponent and non-cone-opponent retinal parvocellular cells were more sensitive to luminance compared to red-green at 1.1 cycles per degree and the cone-opponent cells were more sensitive to red-green compared to luminance at low spatial frequencies. Because the activity of retinal cells influences the activity of higher brain areas, similar spatial tuning to color and luminance can be found in V1. In fact, Thorell, de Valois, and Albrecht (1984) showed that single-opponent neurons (those with narrow spectral tuning and higher responses to full field stimuli) have relatively high responses when presented with chromatic low spatial frequency gratings (blue-yellow due to the spectral tuning of the neuron) and have bandpass tuning to luminance gratings (with highest responses at approximately 0.8 cycle per degree). Conversely, double-opponent neurons (those with similarly narrow spectral tuning but with little to no response to full field stimuli) showed comparable bandpass tuning to both chromatic (red-green in this case) and luminance gratings.

## Conclusions

These two experiments have shown that the perceived contrast (and therefore salience) of stimulus patches in general, and asymmetries in comparisons of bluish/yellowish contrast in particular, vary depending on the spatial pattern of the stimulus. Furthermore, it was demonstrated that although there is an overall increase in perceived contrast with increasing average contrast per area of the patch, perceived contrast also depends on the preferential activation of neurons elicited by the stimulus—different spatial frequency filters, single- or double-opponent neurons. In other words, perceived contrast of stimuli depends not only on the average contrast but also how contrast is distributed within the patch and which mechanisms preferentially process it. This is most probably due to low-level influences caused by basic physiological differences between spatial frequency channels, single-opponent and double-opponent neurons, meaning perceived contrast can be a way of researching neural computations performed by low-level physiological mechanisms. Although there has been much applied research into how color appearance may be translated into luminance appearance for imaging purposes (Bloj, Kersten, & Hurlbert, 1999; Connah, Finlayson, & Bloj, 2007), the mechanisms behind these effects are complex and still not fully understood. Thus they should be explored in more depth and greater detail.

## Open-source practices

Data and analysis code are publicly available to view on OSF at https://osf.io/t5x7n/?view_only=7ffad64e19804e7d9ff6fbfa9447af64.
